# Sustaining LLIN coverage with continuous distribution: the school net programme in Tanzania

**DOI:** 10.1186/s12936-020-03222-8

**Published:** 2020-04-17

**Authors:** Joshua Yukich, Logan Stuck, Sara Scates, Janna Wisniewski, Frank Chacky, Charles Festo, George Kabulika, Kanuth Dimoso, Renata Mandike, George Greer, Naomi Serbantez, Ester Elisaria, Waziri Nyoni, David Dadi, Ikupa Akim, Christian Lengeler, Nick Brown, Hannah Koenker

**Affiliations:** 1 PMI VectorWorks, Center for Applied Malaria Research and Evaluation Department of Tropical Medicine, Tulane University School of Public Health and Tropical Medicine, New Orleans, LA USA; 2 Department of Health Policy and Management, Tulane University of School of Public Health and Tropical Medicine, New Orleans, LA USA; 3 National Malaria Control Program, Ministry of Health, Community Development, Gender, Elderly and Children, Dodoma, Tanzania; 4 Ifakara Health Institute, Dar es Salaam, Tanzania; 5 PMI VectorWorks, Johns Hopkins Center for Communication Programs, Dar es Salaam, Tanzania; 6 U. S. President’s Malaria Initiative, United States Agency for International Development, Dar es Salaam, Tanzania; 7 Swiss Tropical and Public Health Institute, Basel, Switzerland; 8 University of Basel, Basel, Switzerland; 9 A to Z Textile Mills Ltd, Arusha, Tanzania; 10 Nicholas Brown Consulting Ltd., Cardiff, Wales, UK; 11 PMI VectorWorks, Johns Hopkins Center for Communication Programs, Baltimore, MD USA

**Keywords:** Malaria, Schools, Bed nets, Continuous distribution, Long lasting insecticide treated bed nets

## Abstract

Most malaria-endemic countries have struggled in the past decade to establish effective national-scale continuous distribution mechanisms for long-lasting insecticidal nets (LLINs). Since the implementation of the Tanzania National Voucher Scheme in 2004 and mass-distribution campaigns in 2009–2011 and 2015–2016, Tanzania has been committed to finding new and innovative ways of achieving and maintaining universal bed net coverage. Planning for the School Net Programme (SNP) began in 2011 and in 2013, the country piloted a SNP in three regions. Nets were distributed annually to children attending schools in selected primary and secondary grades. Intra-family re-distribution was assumed, and hence the family as a whole, rather than just the children themselves, were the targeted beneficiaries. The programme has since expanded to 14 regions and has seen six rounds of annual distribution. In its fifth year, 3 million nets were distributed at a cost of USD 3.64 per net and USD 0.60 per person-year of protection (including the net). ITN access and use were maintained at a high level (~ 50–75%) over the first 4 years of distribution within selected evaluation areas, even in the absence of a mass distribution event. Net distribution through primary schools has proven to be a feasible and effective strategy for maintaining consistently high coverage in Tanzania.

## Background

Most malaria-endemic countries in sub-Saharan Africa implement mass insecticide-treated net (ITN) distribution campaigns every 3 years to achieve high ITN access, but few have established effective national-scale continuous distribution mechanisms capable of maintaining ITN coverage between mass campaigns or sustaining coverage in the absence of campaigns. Since the implementation of the Tanzania National Voucher Scheme (TNVS) in 2004, Tanzania has been committed to finding new and innovative ways of achieving and maintaining universal bed net coverage. To this end, the country piloted a School Net Programme (SNP) in three regions starting in 2013.

To date, Tanzania and Ghana are the only two countries that have implemented a school net programme on a large scale. The Tanzanian school net experience is notable because of its long duration, large scale and its prominence in the National Malaria Strategic Plan. In order to document this experience, this case study reviews the planning process, implementation, results and lessons learned over nearly a decade of ‘keep-up’ implementation with school nets.

### Bed net distribution in Tanzania prior to the SNP

Between 2009 and 2011, two mass long-lasting insecticide-treated bed net (LLIN) distribution campaigns were implemented in Tanzania with the goal of achieving nationwide universal coverage: the under-five catch-up campaign (U5CC) and the universal coverage campaign (UCC). The two campaigns distributed approximately 27 million LLINs, leading to significant increases in ownership and usage within households [1].

According to the Consensus Statement of the Roll Back Malaria Vector Control Working Group (VCWG), mass campaigns, which allow countries to rapidly reach universal coverage represent a “catch-up” strategy, while strategies which steadily introduce new nets to maintain high ITN access levels after a campaign are defined as “continuous distribution” [2]. Continuous distribution strategies are necessary because nets typically last only two to 3 years in the field, and these lost or worn-out nets need to be replaced to avoid loss of protection. This case study discusses the planning and implementation of an innovative ‘keep-up’ strategy in Tanzania relying on the continuous distribution of LLINs through primary and secondary public schools.

In 2004, Tanzania introduced the first innovative national routine ITN distribution strategy, the Tanzania National Voucher Scheme (TNVS), funded by the first grant round of the Global Fund to fight AIDS, Tuberculosis and Malaria. The TNVS aimed specifically to protect pregnant women (from 2004 onward) and infants (from 2007 onward) with targeted LLIN distribution [3–10]. Pregnant women and mothers of infants received vouchers during clinic visits for antenatal care and measles vaccination. These vouchers were then redeemed for nets at reduced price at participating retailers. The TNVS was discontinued in July 2014 when funding was withdrawn after a donor audit showed serious instances of fraud in some settings using an e-voucher system [11]. As it targeted only pregnant women and infants, the TNVS alone could not provide enough nets annually to maintain universal coverage.

After the cessation of the TNVS in 2014, there was a two-year gap before distribution of free LLINs through routine ante-natal care (ANC) and the Expanded Programme on Immunization (EPI) was instated. Between May 2016 and April 2017, USAID partners distributed nearly 800,000 LLINs through routine health facility-based distribution channels, scaling up on a regional basis and reaching national scale in 2018. The project began with a “Smart Push” strategy in which each health facility received an initial 6-month supply of LLINs. The numbers that each facility received were planned based on patient volume data. Following the “Smart-Push,” facilities report quarterly on the number of nets they distributed and are re-stocked accordingly. The nets were given free-of-charge to pregnant women at their first antenatal care visit, and to children under five when they receive their nine-month measles vaccination.

**Fig. 1:**
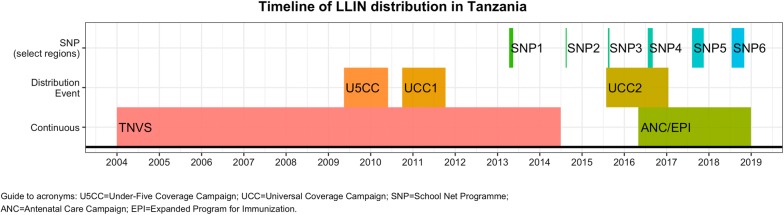
Timeline of LLIN distribution in mainland Tanzania from 2004 through 2018

The history of major bed net distributions in mainland Tanzania since 2004 is shown in Fig. 1. Mass campaigns were first conducted in 2009–2011 (U5CC and UCC), and then again nationally and in non-SNP regions in 2015–2016. Finally, the different school net distributions (described in the following section) are also shown. Before 2004, when this timeline starts, Tanzania already had significant experience with social marketing for ITN, including the KINET, SMITN and SMARTNET projects, and has had a commercial market for bed nets going back to at least the 1990s.

### Development of the school net programme

#### Inception and design

After the mass campaigns of 2009 and 2011, it was widely recognized by stakeholders within mainland Tanzania that in order to maintain universal coverage, additional ‘keep-up’ strategies were needed. To this end, stakeholder meetings were held in June of 2011 in Dar es Salaam, Morogoro, Mtwara, Mwanza and Arusha to elicit input on the operational feasibility of a range of options for additional ‘keep-up’ channels, and to identify potential bottlenecks and barriers to successful implementation at both government and community levels [12]. It was hoped that the strategy would accomplish the following: (1) maintain population use of LLINs at 80% or more, (2) be equitable in terms of access to LLINs, (3) have minimal geographic and temporal gaps in coverage, (4) not oversupply nets to households or be excessively costly and burdensome to manage and administer. It would also put some degree of responsibility on households to acquire nets, either through effort (e.g. travel, self-registration) or through paying a small portion of the cost of the net, as was done through the TNVS. The system would also encourage fair competition among manufacturers to improve quality and reduce costs. In an ideal system, a choice of nets (in terms of size, fabric, and colour) would also be available to the consumer [12].

In order to leverage the TNVS platform, distribution of vouchers for free or highly reduced price LLINs to school children in primary and secondary public schools in combination with the TNVS was identified as the best strategy to maintain universal coverage over a range of other options [12]. At the time, enrollment in primary school in Tanzania was generally high, with all but two regions reporting net enrollment rates greater than 90% [13]. Roughly 30% of the population of the country was between five and 15 years of age and was thus eligible for primary school enrollment and at least 62% of households had current resident of primary school age [12, 14]. Stakeholder discussions confirmed that beneficiary identification was clear and simple as only students enrolled in school would be eligible. It was assumed that teachers and school health officials could capably facilitate distribution and monitoring. Stakeholders noted that since households already needed to pay for school fees and uniforms at the beginning of the school year that the additional expense of an ITN through a subsidized voucher with co-payment could pose a barrier to school enrollment and might be expected to reduce voucher redemption rates. On the other hand, receipt of vouchers could incentivize enrollment and thus potentially boost enrollment levels. Ultimately, only the alternative with free net distribution was piloted and eventually expanded. After the TNVS was discontinued, use of a voucher-based SNP was no longer considered a short-term viable approach and a SNP with direct distribution of ITN to school children was piloted instead. Decisions regarding the design, scope and scale of the pilot programme were made by the Tanzania NMCP, especially the ITN cell embedded in the NMCP, and supported by the Swiss Agency for Development and Cooperation (SDC) in collaboration with donor partners and based in part on the results of the initial projections and results of the multi-stakeholder collaboration meetings detailed in Koenker et al. [12]. During the initial planning that resulted in the SNP, projections for the full keep up system (combined TNVS and School Nets) only considered the voucher-based SNP option [12]. Therefore, we retrospectively consider these as the projections of cost and coverage for the SNP [12]. The School Net Programme was formed as a programme for free LLIN distribution annually to targeted households with school children as last-mile distributors. The attractiveness of the school net distribution strategy was that (1) it has a very clear and simple identification strategy, (2) the potential to reach 85% of the population when combined with the TNVS (which was expected to only reach 45%), and (3) that it could be clearly monitored.

#### Pilot testing and expansion

**Fig. 2:**
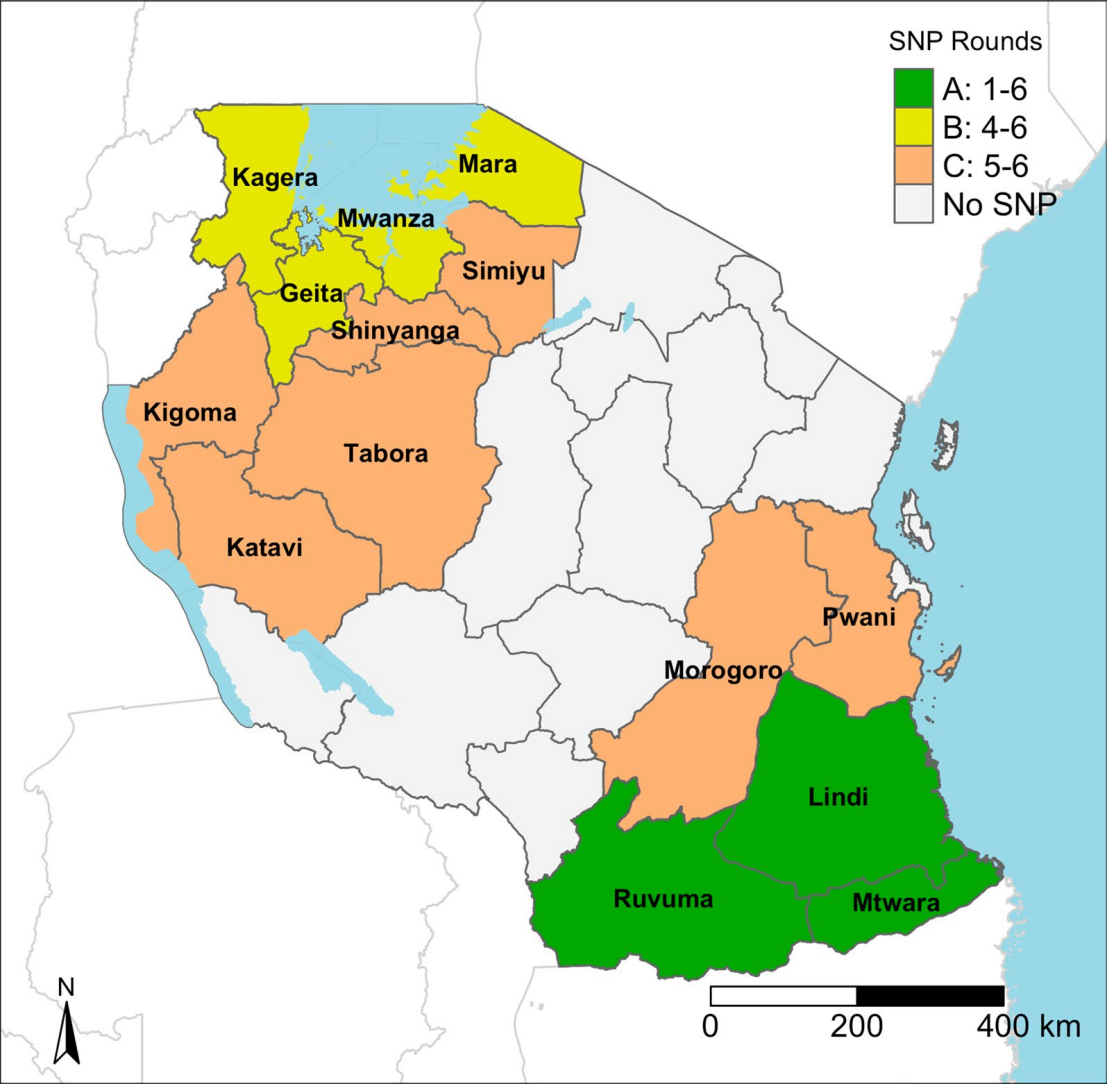
Map of SNP implementation areas

The School Net Programme (SNP) was piloted in 2013 in Southern Tanzania (Lindi, Mtwara, and Ruvuma regions) by the Ministry of Health, Community Development, Gender, Elderly and Children (MoH-CDGEC) in partnership with the Ministry of Education and Vocational Training (MoEVT), Funding came from the U.S. President’s Malaria Initiative (PMI) through the United States Agency for International Development (USAID) and the SDC. Further rounds of issuing LLINs within the SNP were funded by USAID/PMI, and implemented by a consortium of partners that varied over time (including: RTI International, Tanzania Red Cross Society, PSI, JHUCCP) and was led by the National Malaria Control Programme (NMCP) with support from the Swiss Tropical and Public Health Institute and SDC support. To date, the SNP has been rolled out over six annual rounds (SNP1-6) in a step-wise fashion across 14 regions of Tanzania (Fig. 2). The first three rounds of the SNP occurred in the three regions of Lindi, Mtwara, and Ruvuma (SNP Area A). The SNP expanded beginning with round four adding the four regions of Mwanza, Mara, Kagera and Geita (SNP Area B). In round five, the SNP was expanded again to add the seven regions of Katavi, Kigoma, Morogoro, Pwani, Shinyanga, Simiyu, and Tabora (SNP Area C).

In the SNP, one LLIN was distributed to each child enrolled in an eligible grade (originally primary and secondary school students). When the children received a net, they were told to bring it home and that it should be used according to needs in the household. They were also told that they may give the net to someone else who needed the net if it was not required in their household. Grade eligibility differed between rounds and regions. It was determined by the NMCP based on annual estimation of both needs for LLINs and resource availability. Broadly, SNP1-3 delivered ITNs to four to six classes each year. Regions that had received the 2015–16 mass campaign and then began school distribution in SNP4 or SNP5 had one to four targeted classes in their first year—with more classes the longer the time elapsed since the most recent mass distribution campaign.

After SNP2, distributing nets to secondary schools was not deemed a strategic priority due to the low enrollment rates in secondary schools, and so distribution was limited to primary school students from SNP3 forward. Enrollment levels in secondary schools in Tanzania are low relative to primary schools and may have decreased in recent years. During the same time, primary age enrollment increased from 85.7% to 91.3%, most notably after the Government of Tanzania abolished primary school fees for the 2017 school year, prior to SNP5.

#### Projection of coverage and cost

At the time of the initial planning of the SNP programme, predicted net ownership and usage levels were modelled using the NetCALC software developed on a Microsoft Excel platform. The version used for the original modelling is now obsolete but updated versions remain available (https://www.vector-works.org/resources/netcalc-planning-tool/). Cost modelling, conducted during planning, used data from various sources, including the NMCP of Tanzania, published literature, and financial records and reports of implementing partners of the NMCP to predict the future financial cost of the planned net distribution programme and other options. Commodity costs for nets were assumed not to vary over time. The costs of voucher distribution for school distribution were assumed to be similar to those under the TNVS at that time. Distribution of nets freely directly to students rather than through the use of a voucher assumed the same costs as had been achieved in past campaign distributions [12].

The original coverage and use modelling assumed that approximately 73% of households, representing 85% of the population, included a currently pregnant woman, an infant (under 1 year) or a current student [14]. A that time, there were approximately 8.4 million primary school enrollees in Tanzania during the 2010 academic year.

Delivery of school net vouchers to 90% of primary school enrollees with an 80% redemption rate was expected to result in the delivery of approximately 6.2 million LLINs per year into households in Tanzania, falling short of total net replacement need estimated at 7.9 million LLINs per year [12]. Modelling the delivery via vouchers to all primary school students each year was estimated to require 56.7 million LLINs over 10 years at a total cost of $389 million, resulting in 73% of all person-years protected (proportion of person-years at risk in which consistent ITN use was expected to occur) with a cost of $1.32 per person-year-protected (PYP). If free nets were provided instead, due to slight gains in delivery success, a need of 67.3 million LLINs was estimated at a total cost of $466 million, resulting in 83% of all person-years protected at a cost of $1.41 per person-year protected (all costs are inclusive of the costs of the nets).

In 2013, a combination of the TNVS (targeting pregnant women and infants) and LLIN distribution through schools was thought to be an attractive combination of ‘keep-up’ mechanisms to reach the yearly net replacement need of 7.9 million LLINs. Roughly 9% of households had one to three children between the ages of one and five, and thus would have recently been eligible for the TNVS within recent years. These households would also be expected to move into the targeted category for SNP as the children reached school age. This left about 18% of households untargeted by the combination strategy, even over a multi-year window. Hence, this work provided the basis for assuming that a very substantial part of the population could be reached via the SNP.

**Fig. 3:**
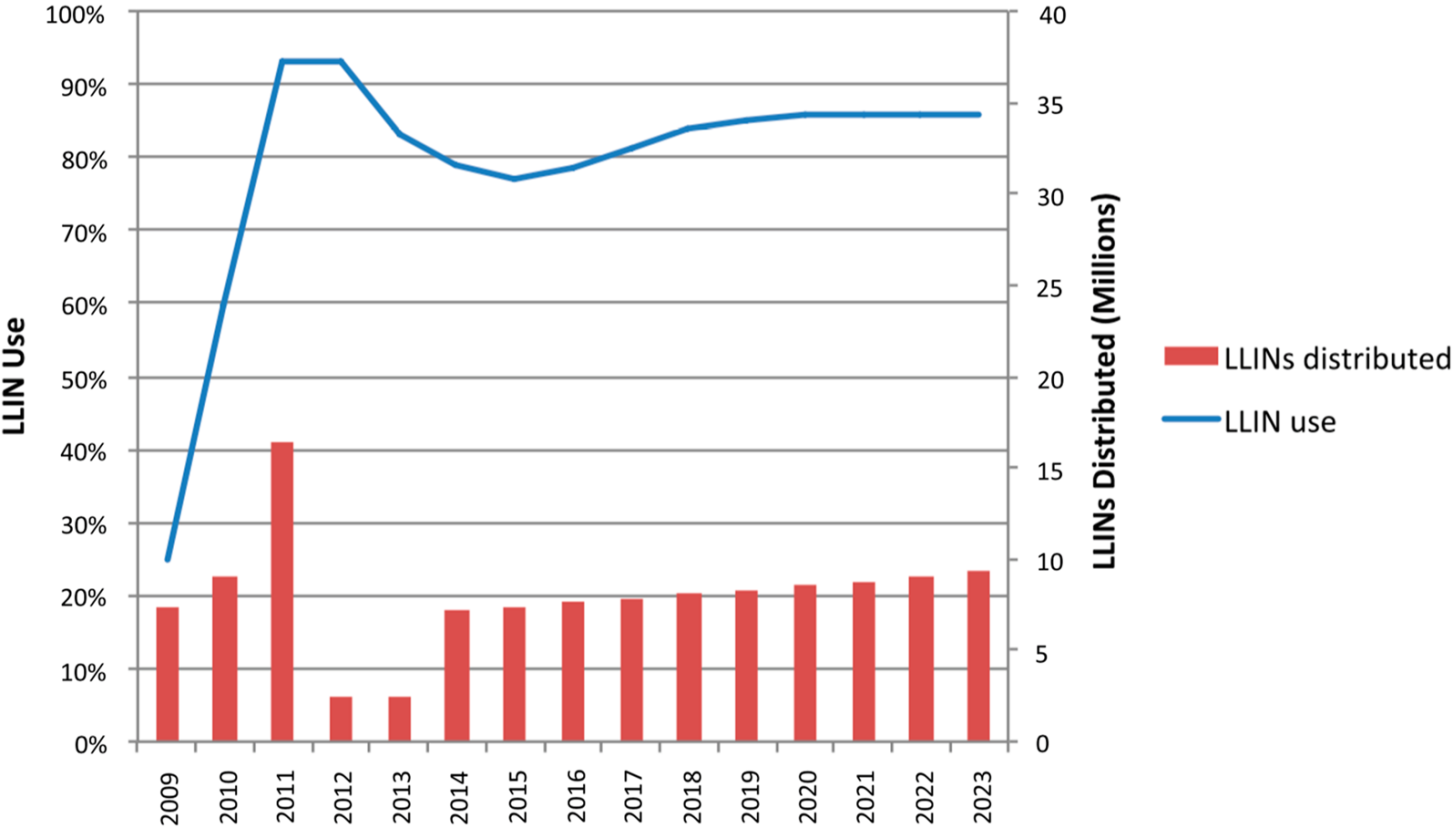
Projections for the combination of TNVS and school voucher distribution [12]. The years 2009–2011 show a rapid increase in use due to the modelled effect of the mass distribution carried out in that period

Figure 3 illustrates the modelled combination of the TNVS with school net vouchers distributed each year in Standards one, three, five, and seven (primary school) and Forms one and four (secondary school), which was the scenario most similar in the planning phase to what was actually implemented in Tanzania during SNP1. Targeting pregnant women and infants through the TNVS and students in these selected school grades was expected to lead to sustained use of ITNs by the population as a whole at about 82%. Over ten years the combination of TNVS plus school net voucher distribution was estimated to require 65.4 million LLINs at a total cost of USD 449 million for the entire country, and was predicted to protect 82% of total person-years at risk, at a cost per PYP of USD 1.34, and a per net cost of USD 6.87 (including the net itself).

### Performance of the ‘keep-up’ strategy

#### Coverage

To evaluate the coverage achieved by the SNP, annual rounds of a household survey were carried out in four districts of Tanzania, beginning in 2013. Two Southern districts, Mtwara Urban and Nachingwea, were located in the pilot regions and made up the intervention arm of the evaluation (SNP Area A in Fig. 2). The other two districts, Chato and Sengerema, were initially located in non-implementing Lake regions and were thus intended to make up the control arm of the evaluation (SNP Area B in Fig. 2). However, after the second survey round, the second UCC was rolled out in the control areas. Further, the SNP was expanded to include the control districts after the third survey round. Details of the evaluation are published in additional manuscripts [15] (Stuck, pers. commun.).

#### Ownership, access, and use

**Fig. 4:**
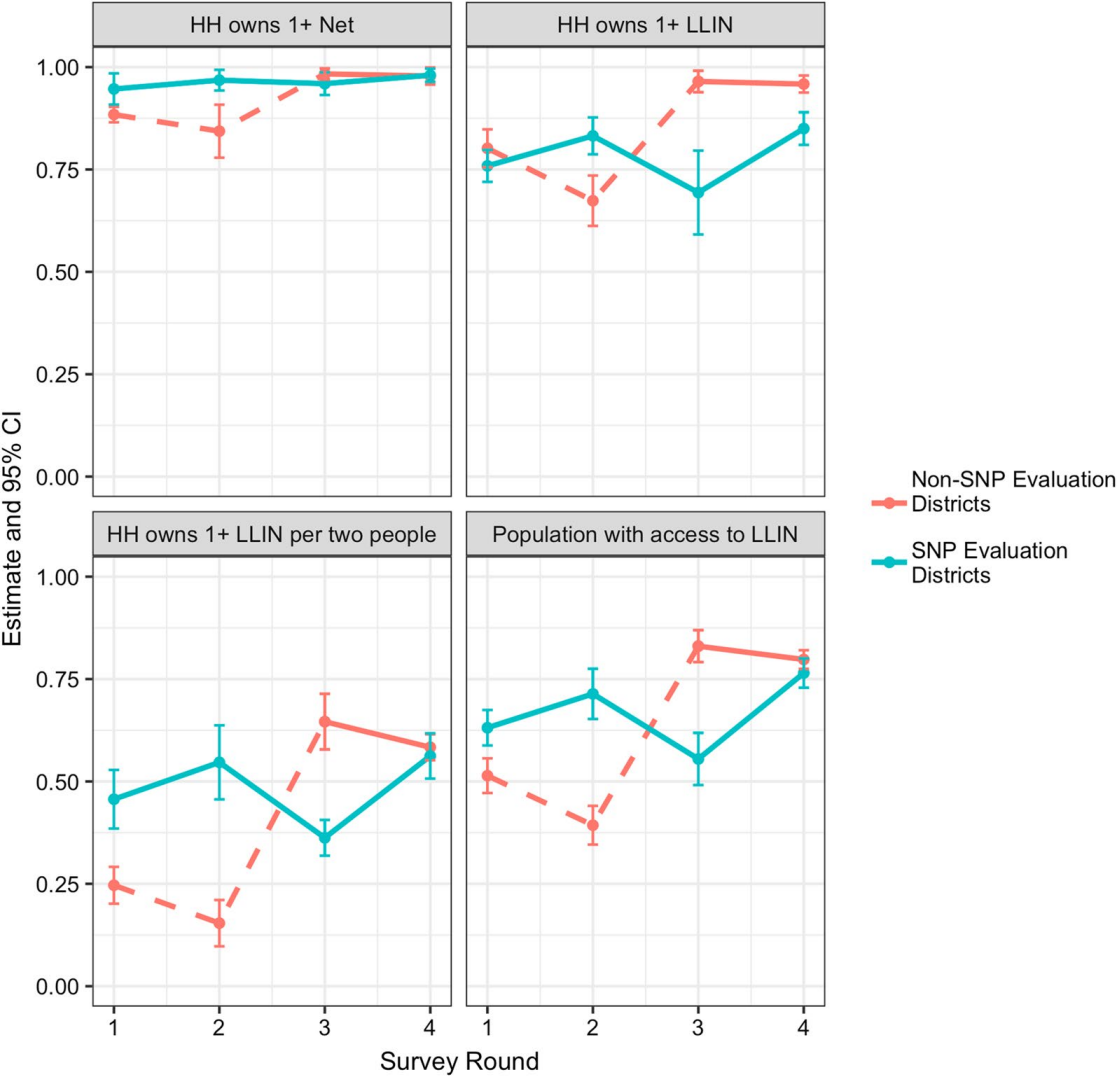
Household ownership of one (any bed net and LLIN), one LLIN per two persons, and population access to LLIN as measured in four household surveys. Non-SNP Evaluation estimates represent samples in areas with no SNP before the first three surveys (but a mass coverage campaign between survey rounds 2 & 3), while SNP Evaluation Districts estimates represent areas with full SNP rounds implemented prior to each survey. Dashed lines show time periods within the non-SNP evaluation districts prior to roll out of SNP in these districts

**Fig. 5:**
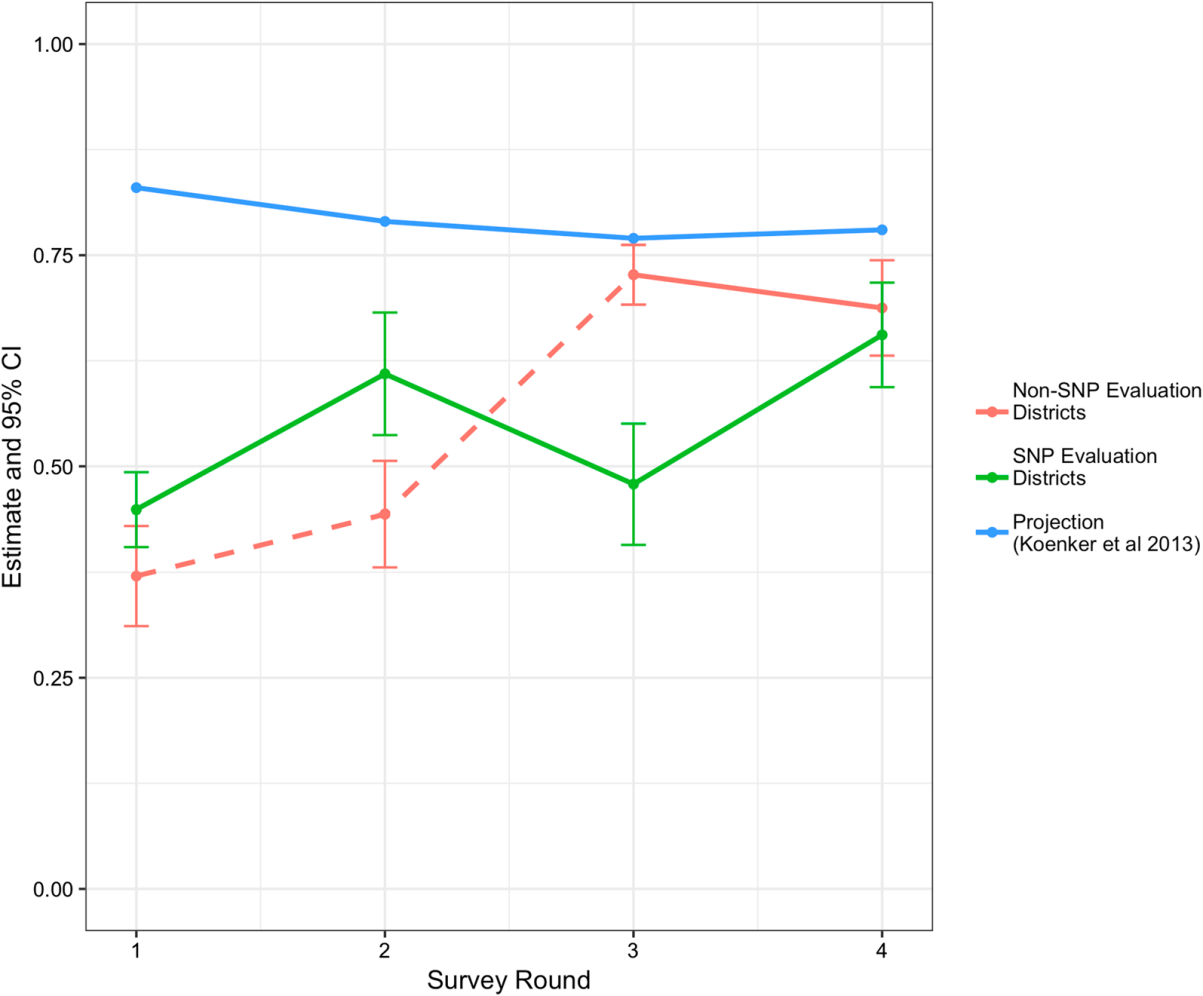
Observed ITN use vs projected ITN use from original NetCALC Model [12] (Stuck pers. commun.). Dotted red lines shows non-SNP districts before SNP was expanded to include them. A mass campaign was conducted in the non-SNP areas between Survey Rounds 2 and 3

During a 4-year evaluation of the programme, ownership, access, and use indicators were maintained or even increased from baseline levels within intervention districts (Fig. 4) (Stuck, pers. commun.). Over the evaluation period in Southern Tanzania, LLIN use in the total population increased from 44.9% (95% CI 40.5–49.3) to 65.6% (95% CI 59.4–71.8; Fig. 5) based on measurements in two evaluation districts (Stuck, pers. commun.). While the final use estimate fell short of the projected 80%, the starting point was much lower than anticipated (~ 45% actual vs > 80% anticipated). Several factors likely led to the discrepancy between predicted use and actual use. One primary factor was the delay in implementation of the SNP after the first UCC campaign, which was implemented in the Southern Zone in 2010. While original modelling expected the SNP to begin in the following year, the SNP did not start until the third year after the campaign, when the availability of nets in the three targeted Southern regions had already fallen dramatically. Secondly, the discontinuation of the TNVS in 2014 and a two-year delay in the resumption of routine distribution through ANC/EPI also resulted in significantly fewer nets than expected being delivered in the targeted southern regions. A third important factor was that initial modelling assumed that the median LLIN half-life was 3 years, which was a common assumption at the time. Current information based on rigorous field studies suggests that median half-life for LLINs in mainland Tanzania is closer to 2 years [16]. This longer lifetime assumption results in dramatically higher coverage predictions than would have resulted from using the Tanzania-specific, shorter estimates that are now available. Finally, the modelling method at the time utilized a simple assumption that use would be approximately seven percentage points lower than ownership of at least one LLIN. As it turned out, on average in Tanzania, there were 7–11 percentage points between population level access and individual use at the national level over the past four MIS/DHS surveys, but 17–22 percentage points between household ownership of any ITN and individual ITN use. Since this time, many additional analyses of the relationship between net use, ownership and access have been conducted, showing that population ITN access is a better indicator of ITN “coverage”, and that net use is more accurately modelled in relation to population access [17, 18]. Nonetheless, by the end of the SNP evaluation, net use indicators had been at least maintained, if not increased, from their baseline levels as was part of the primary original goal of the SNP.

#### Reach of the SNP (household level eligibility and enrollment)

Over the four evaluation rounds, an average of 61.9% (95% CI 59.0%, 65.0%) of all households housed a primary or secondary school-aged child and 40.0% (95% CI 37.2%, 43.0%) of all households housed a child enrolled in an eligible grade. By the end of the evaluation, 52.5% (95% CI 48.3–56.7) of households owned at least one net obtained from the SNP. Pooling the data across all four rounds, 34.0% (95% CI 30.7%, 37.4%) of all surveyed households in SNP areas owned a net distributed through the SNP during the previous SNP round (42.2% of the surveyed population of these areas lived in a house with a SNP net obtained during the previous SNP round). This result indicates that each round of the SNP reached approximately one of every three households, but that different households were reached each year.

From SNP1 onward, Social and Behaviour Change Communication (SBCC) activities were intentionally designed to encourage families to give extra nets to relatives or neighbours to improve equity of ITN access. Gifting of SNP nets outside a given household was uncommon immediately after the first distribution (0.9% of all SNP nets) but increased to 15.4% of all SNP nets by the fourth round of distribution. With time and supportive communication, this process could potentially represent a pathway by which the SNP could reach non-target households.

### Cost

#### SNP

Formal costing following standard practices was conducted during the third and fifth rounds of SNP distribution [19]. SNP3 delivered nearly 500,000 LLINs through schools in three regions at a cost of 9.48 USD per net distributed (1.58 USD per PYP) and SNP5 delivered slightly more than three million LLINs through schools in 14 regions at an economic cost of USD 3.64 per net distributed (USD 0.60 per PYP) in 2017 (including the price of the net). Of the total costs, approximately USD 5.96 was for distribution and the remainder (USD 3.52) related to the cost of the net itself in SNP3, while approximately USD 1.58 was for distribution and the remainder (USD 2.06) related to the cost of the net itself in SNP5. For both SNP3 and SNP5, the most significant cost driver in financial terms was the price of LLINs, though LLINs accounted for less than 50% of total cost in SNP3. The second most important cost drivers were the expenditures on personnel and fringe, school data quantification and validation, and supervision for SNP3 distribution and net transport and personnel and fringe for SNP5 distribution. The economic cost per net distributed in SNP5 was substantially lower than SNP3, largely due to cost-cutting measures introduced at the national and local levels. From SNP3 to SNP5 there was a significant shift of focus from local government level to higher-level engagement by obtaining student enrollment data from the central government and working closely with national-level representation to develop new tools. At the same time, data collection and reporting within the central government was strengthened and incorporated into SNP5 by utilizing existing infrastructure such as the Basic Education Management Information System (BEMIS). Projections for the combined TNVS/school voucher programme indicated that 65.4 million LLINs would be distributed at a cost per PYP of USD 1.34, and a per-net cost of USD 6.87 (including the price of the net). SNP3 delivered nets at a higher cost per net and per PYP than was initially predicted, by SNP5 much lower costs per net and cost per PYP than original projections were being achieved [12]. Given that the later rounds of SNP delivered substantially more LLINs than the early rounds and at a lower cost, the total cost of the programme over a ten-year period would be expected to be much lower than initially projected due to a combination of falling commodity costs for LLIN and more efficient distribution. Further gains might be made if distribution costs could be further reduced; as newer more expensive ITNs come online such gains may be necessary to contain costs at recent levels.

#### ANC/EPI distribution

The costs of routine distribution had originally been assessed in the TNVS prior to the roll-out of the SNP, and predictions of the cost of the TNVS during the SNP intervention were based on this [7]. The TNVS was discontinued in 2014 and by 2016/17 this element of the ‘keep-up’ strategy was replaced by routine free distribution of LLIN at ANC/EPI clinics. A formal economic and financial costing of the ANC/EPI distribution was conducted in 2018 and estimated the financial cost per ITN distributed through ANC/EPI to be USD 6.04 per net, not including domestic government contributions (such as ANC/EPI clinic staff salaries and storage space), and USD 7.50 when these were included. These translated into a cost of approximately USD 1.25 per person-year of protection which is similar but slightly lower than the prediction from the original planning period (all costs include the cost of the net).

### Challenges and lessons learned

#### Weaknesses in design

*Gaps in reach.* Excluding secondary school students from the targeted population after SNP2 meant that households with only older children (in addition to those with none) were no longer reached by either SNP or ANC/EPI distribution—except through the relatively uncommon event of inter-household redistribution of nets. These included households with older adults whose children had grown past school age, households made up of young families who did not yet had children, and single or transitory workers living in shared housing. While such households were likely to benefit from the community effects of nets given the high levels of usage and ownership in SNP areas, questions of equity and reach need to be considered. Tanzania’s current National Strategic Plan has a commercial sector component intended to boost ITN sales so that all households would be able to purchase an ITN when they need a new one, but unfortunately, most purchased nets are untreated [20]. As the programme expands, it will be critical to determine if local variation in school enrollment will result in compromised coverage effects in some parts of the country, and whether varying the number of targeted classes will be able to mitigate this.

*Overestimate of net lifetime.* While predictions of cost per person-year of protection and per net distributed from before the initiation of the SNP were relatively accurate, predictions of use and ownership were somewhat optimistic. One important reason for overestimation was a reliance on an overly optimistic estimate of net lifetime. The lower observed coverage highlights the importance of, at a minimum, gaining a better understanding of LLIN lifetimes to drive procurement quantifications and plan for future distribution to reach pre-determined coverage targets. Overly pessimistic estimates of net lifetime will lead to oversupply of nets while overly optimistic assumptions will result in insufficient coverage and potentially insufficient protection.

#### Challenges in implementation

*Timing of first SNP and interruption in routine delivery*. The TNVS ended in July 2014 after an audit uncovered fraudulent activities by health facility staff, retail outlets, and sales representatives [11]. After a two-year gap in distribution targeted at pregnant women and infants, distribution to these populations was slowly reinstated via free LLIN distribution through ANC and EPI. This gap in routine delivery led to significant undersupply of nets when compared to original planning for the SNP in 2011. Likewise, the 2011 Keep Up Strategy projected SNP to begin in 2012, while it did not start until mid-2013, nearly 3 years after the last mass campaign in the three targeted southern regions.

*Large number of distribution points.* The large number of schools and hence distribution points for the SNP posed a major operational challenge. The project began with 2337 primary and secondary school distribution points and expanded to 9535 primary school distribution points across the 14 targeted regions by the fifth round of SNP. The operational scope is similar to mass campaigns, which had 2668 distribution points in the 2010-2011 UCC in the three originally targeted southern regions, and 11,717 issuing points for the UCC in the 14 regions currently targeted for SNP. SNP shared some operational challenges with other large-scale net distributions, such as identifying the optimal cascade training design, having consistent cellular network service to facilitate mobile-money payments for trainees and supervisors, and data entry errors and delay as reports were aggregated. Sensitization meetings with local officials and trainings for various actors were relatively intense in the first rounds; these were reduced or eliminated entirely in subsequent rounds as the programme became institutionalized.

During SNP1, LLINs were stored for several days so that all schools could distribute nets on the same day, region-wide; this was eliminated in later rounds to avoid storage and security costs. LLINs were given to teachers in targeted classes in SNP1. This practice, along with paying allowances for issuing and reporting, was eliminated in later rounds as the time involved was minimal and recognized as part of teachers’ job duties. Ward Education Coordinators (WECs) initially visited schools to collect enrollment data, which was cumbersome to manage. School data validation visits were employed in SNP2 and SNP3, but eliminated, along with the WEC role, for SNP4 when the President’s Office Regional Administration and Local Government offices (PO-RALG) established the Basic Education Management Information System (BEMIS) to track enrollment data. Data from the BEMIS were used to establish LLIN quantifications and LLIN transport plans. Logistics were also streamlined over time-in the first rounds, LLINs were delivered to the district councils and re-bundled for separate onward transport to various schools. Switching to a ‘mobile warehouse’ strategy where larger trucks dropped off nets at schools along pre-identified routes reduced costs, as did switching in SNP4 from offloading, storing, and reloading at regional warehouses to a ‘cross-docking’ system whereby LLINs were received virtually by regional authorities, but offloaded directly to the smaller ‘mobile warehouse’ trucks. Starting in SNP5, transport was done by a third-party private logistics firm, with delivery timeframes and confirmations sent by SMS to district council officials and implementing partners. The ministry of local governments (PO-RALG) increased their involvement in the activity over time, given their oversight of local-level health and education activities. As SNP expanded to additional regions, the timeframe of the annual activity lengthened. LLIN shipments were planned to arrive in two to three phases, scheduled around examination periods and vacations. In limited cases, deliveries occurred after exams when Standard 7 students had already left school. Timing distribution around holidays, national exam schedules and local events also poses a planning challenges, as school distributions must align well with school calendars which can vary significantly even within small areas.

*Acceptability to stakeholders and beneficiaries*. The reactions of stakeholders and beneficiaries has been largely positive, with students and teachers generally looking forward to the SNP. In parallel, local government involvement and leadership has grown over time [21]. Through the planning discussions for SNP1-3, NCMP and PO-RALG centralized data and improved the systems, allowing both government and implementing partners to access the data at a central level. As a result of this collaboration, the SNP helped PO-RALG formalize data management related to enrolment, hence offering an appreciated service. Since SNP4, the consensus was that work was easier—classes were preidentified without costly and time-consuming visits to collect or verify data, and distribution occurred the same day nets were delivered. Others also felt that the programme reduced malaria cases in student populations. SNP has also led PO-RALG, the MoEVT and the MoH-CDGEC to coordinate more closely, an important development towards multi-sectorial malaria control activities.

There are also ongoing concerns with some elements of the programme, especially with regards to groups that are missed by the SNP. This includes dissatisfaction among students enrolled in schools but not in eligible classes in a given year, especially those in the youngest classes and the pre-school classes (kindergarten classes). It also includes students enrolled in schools that may be missed because they are not part of the regular Ministry of Education System, such as schools for students with special needs, which may not be identified through the regular school quantification system. Extra care must be taken in future SNP-like programmes to ensure that such schools are enumerated and included. In addition, in some early years, teachers were included as beneficiaries (hence they received a net), but in more recent years they have only been programme participants. The teachers repeatedly reported the desire to be included in the programme as beneficiaries [21].

*Quantification of net needs.* In addition to the original modelling that explored the theoretical framework for the SNP, for each SNP round, NetCALC was used to conduct quantification before net procurement and deployment. Several challenges and some lessons learned through this process have led to improvements in the overall quantification modelling process. The original NetCALC software could only predict ownership of at least one net at the household level (though this was modified to also predict use for the initial SNP planning) [12]. Subsequent versions of the modelling software were updated to formally predict ITN population access. During SNP1-3, ownership of any ITN was the target used during quantification, while population ITN access was used to quantify net needs after SNP4, resulting in better planning alignment with national strategic goals. Regardless of the target indicator used for quantification, net lifetime was an important parameter which needed considerable revising over time and still require local monitoring [16, 17]. The use of an Excel-based tool limited options for an effective, error-free workflow and created opportunities for data entry error as the programme expanded in scope and became more complex. A NetCALC algorithm in software that is more reproducible has become available and may be useful moving forward [22]. Higher resolution data in time and space, such as was available due to routine evaluation was helpful for quantification, as LLIN needs could be locally recalibrated each year and the strategy adjusted based on these data [15]. This was especially true in the early rounds of SNP, when assumptions about the reach of the strategy were confronted with the realities of the three-year gap since the previous mass campaign and the discontinuation of the TNVS. Given the variations in enrolment by region, as well as different starting years and starting levels of ITN access, good monitoring will continue to be important to track ITN access on a regional basis, gathering data, and adjusting net quantities to maintain high rates of ITN access.

## Conclusion

The Tanzania School Net Programme was able to maintain LLIN ownership, access and use in the absence of a mass distribution over a period nearing a decade since the last mass campaign in three southern regions of Tanzania. LLIN population access in these areas was comparable to regions with recent mass distributions in the most recent nationwide surveys (70–80% in the 2017 MIS). The SNP successfully reached households with school-age children but left a substantial number of households that did not have school-enrolled children without sufficient ITNs, thus requiring additional channels such as ANC/EPI for long-term coverage maintenance. A mechanism to reach households without children is still lacking leading a substantial portion of the population without a direct avenue to access ITNs.

Predictions of cost and coverage leading to the initial deployment of the SNP were relatively accurate but compromised by incomplete understandings of LLIN lifetimes, late start of the pilot SNP, and limitations of the original NetCALC model. In addition, coverage was initially negatively impacted by the discontinuation of the TNVS in 2014, which had been factored into the original planning.

Despite these setbacks to the SNP, distributing LLINs through primary schools has proven to be a viable strategy as an alternative to repeated mass campaigns in Tanzania and has thus been expanded to about half the country. Finally, the combination of the SNP with other preventive measures, as defined in the National Malaria Strategic Plan, will require future assessment to understand if they continue to serve the role of providing sustainable LLIN coverage, reductions in malaria burden and progress towards a malaria free Tanzania.

## Data Availability

Data sharing is not applicable to this article as no datasets were generated or analysed during the current study.

## Abbreviations

ANC: Antenatal care
BEMIS: Basic Education Management Information System
EPI: Expanded Programme on Immunization
ITN: Insecticide treated bed net
LLIN: Long lasting insecticide treated bed net
MIS/DHS: Malaria indicator survey/demographic and health survey
MoEVT: Ministry of Education and Vocational Training
MoH-CDGEC: Ministry of Health, Community Development, Gender, Elderly and Children
NMCP: National Malaria Control Programme
PMI: US Presidents’ Malaria Initiative
PO-RALG: Prime Ministers Office of Regional and Local Government
RBM: Roll back malaria
SBCC: Social and behaviour change communication
SDC: Swiss Development Corporation
SNP: School net programme
TNVS: Tanzania National Voucher Scheme
U5CC: Under five coverage campaign
UCC: Universal coverage campaign
USAID: United States Agency for International Development
USD: United States Dollar
VCWG: Vector control working group
WEC: Ward education coordinator
